# A Rare Case of Subglottic Malignant Lymphoma Requiring Emergency Tracheostomy

**DOI:** 10.7759/cureus.94893

**Published:** 2025-10-18

**Authors:** Teru Kamogashira, Kazuka Arimoto, Megumi Kishimoto, Kazuki Miyano, Shinichi Ishimoto

**Affiliations:** 1 Department of Otolaryngology, JR Tokyo General Hospital, Tokyo, JPN

**Keywords:** cd5-positive malt lymphoma, extranodal marginal zone lymphoma, larynx, low-grade b-cell malignant lymphoma, subglottic, tracheostomy

## Abstract

Primary malignant lymphoma originating in the laryngeal tissue is rare. A 61-year-old woman was admitted to our hospital with hoarseness and mild dyspnea for three weeks. The fiberscopic examination revealed slight edema around the vocal cords. Despite four weeks of inhaled steroid therapy, progressive worsening of the subglottic lesion led to increasing airway obstruction, and due to diagnostic uncertainty and the risk to the airway, emergent tracheostomy was performed. Fiberscopic findings through the tracheostomy stoma revealed multiple tumors around the anterior tracheal wall and a submucosal bulge around the posterior tracheal wall in the subglottic area, so biopsies from both areas were performed. Histological examination revealed a low-grade B-cell lymphoma (probable CD5-positive mucosa-associated lymphoid tissue (MALT) lymphoma, unclassifiable). As a further biopsy would have required general anesthesia and might still have yielded insufficient tissue for definitive subclassification, treatment was initiated for an unclassifiable low-grade B-cell lymphoma, with a plan to perform a repeat biopsy at recurrence for diagnostic confirmation. The patient subsequently received chemotherapy with bendamustine and rituximab and has sustained a complete response for seven years.

## Introduction

Primary laryngeal lymphoma is a rare malignant tumor arising from lymphoid cells in the larynx, accounting for less than 1% of all laryngeal malignant tumors, and laryngeal lymphomas account for less than 1% of all extranodal lymphomas [1-4]. Since its first report in 1934 [5], fewer than 100 cases have been documented [1,6-8]. Unlike the far more common squamous cell carcinoma [9], which originates from the epithelial lining, laryngeal lymphoma is uncommon because the normal larynx lacks native lymphoid tissue, making primary lymphoid neoplasms pathophysiologically exceptional [10,11]. Histologically, the most common subtypes are plasmacytoma, mucosa-associated lymphoid tissue (MALT) lymphoma, and diffuse large B-cell lymphoma [12,13]. Among the laryngeal subsites, the supraglottic area is most frequently involved (46.5-76.5%), followed by the glottis (13%) and the subglottic area (10.5%) [1,14,15]. The age of onset for laryngeal lymphomas ranges from four to 81 years, with a mean age of 64.2 years [1].

The initial symptoms of primary malignant lymphoma of the larynx, such as hoarseness, throat discomfort, mild dyspnea, or cough, are highly nonspecific and frequently overlap with benign conditions such as acute or chronic laryngitis, vocal cord polyps, or reflux laryngitis, making suspicion of malignancy less likely at an early stage [16,17]. Due to the rarity of the disease, clinicians often do not initially suspect it, which can lead to missed diagnoses in primary care [18,19]. Lymphomas often grow submucosally; therefore, superficial biopsies may fail to yield sufficient tumor tissue [20]. Deep or extensive sampling may require biopsy under general anesthesia, and concerns about invasiveness or potential complications can delay repeat biopsy. Even on imaging (CT/MRI) and laryngoscopic examination, lymphomas have been reported to be difficult to distinguish from inflammatory thickening or benign tumors, so definitive diagnosis is often not possible based on imaging alone [17,21]. Lymphomas arising in the subglottic region are particularly rare, with reported cases being very limited [22,23]. These lesions often cause airway narrowing and respiratory symptoms, and their clinical presentation frequently resembles scar-related stenosis or chronic inflammation, making misdiagnosis common.

We report a rare case of CD5-positive low-grade B-cell lymphoma in the subglottic region, representing an unusual immunophenotype at this site and resulting in airway stenosis necessitating tracheostomy.

## Case presentation

**Figure 1:**
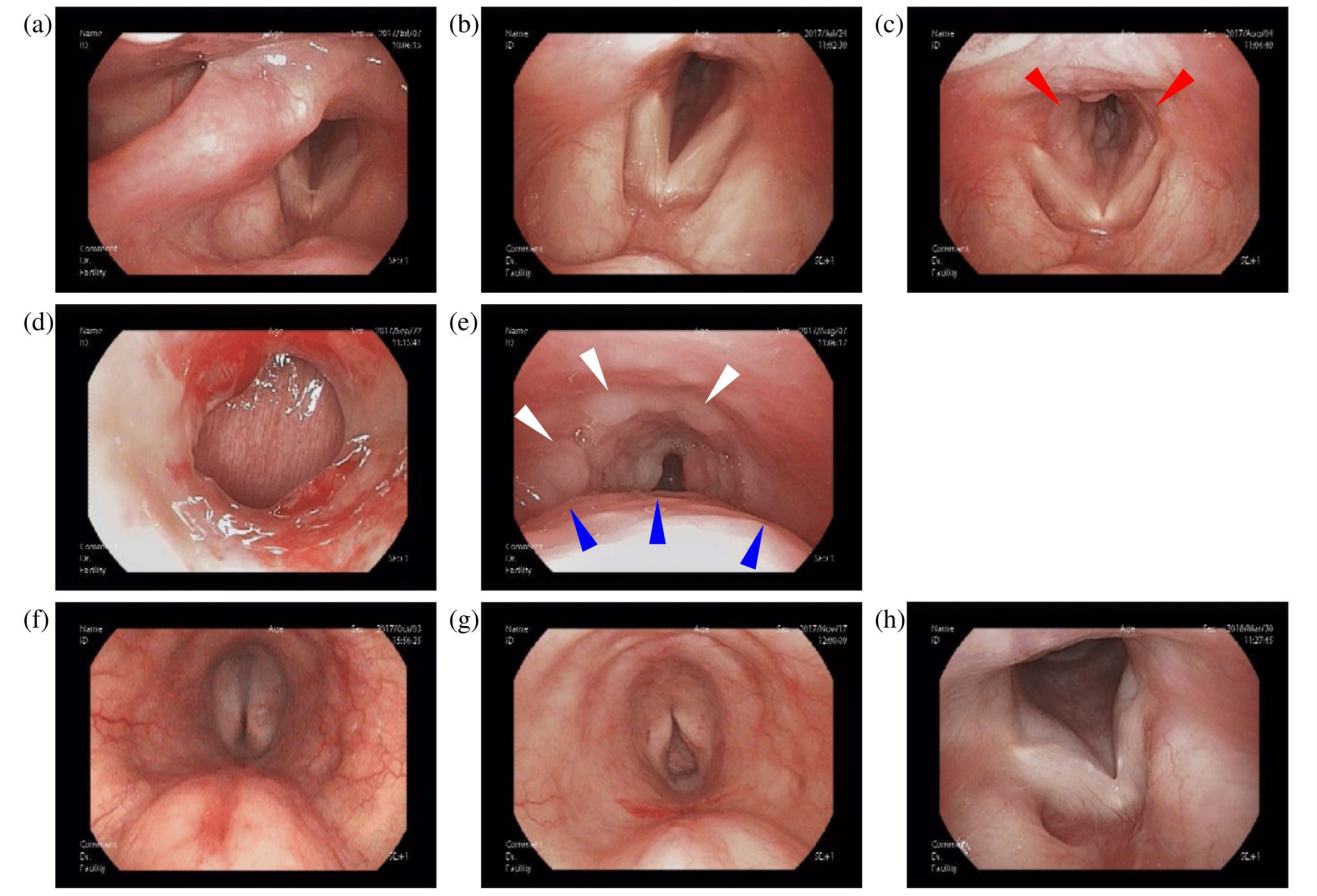
Endoscopic images (a) At the first visit; (b) After two weeks; (c) After four weeks. Red arrows indicate slight edema around the vocal cords and subglottic areas; (d) Tracheostomy site; (e) Subglottic view through the stoma (shown in d) prior to chemotherapy. White arrows indicate multiple tumors along the anterior tracheal wall. Blue arrows indicate a submucosal bulge of the posterior tracheal wall; (f,g) Subglottic view through the stoma (shown in d) following the first (f) and second (g) courses of chemotherapy; (h) One year after chemotherapy.

**Figure 2:**
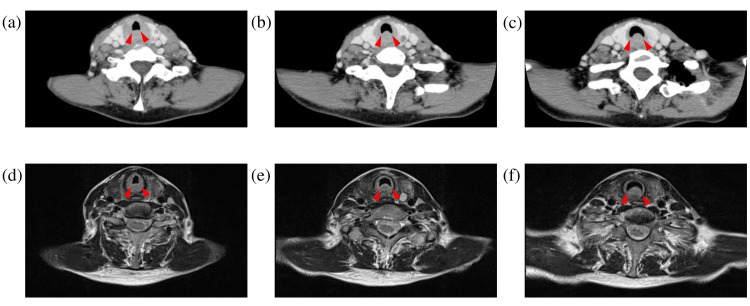
CT and MRI images (a–c) Contrast-enhanced CT scans of the tumor; (d–f) T2-weighted MRI scans of the tumor. Red arrows indicate the bulging posterior tracheal wall and the iso-dense circumferential lesion extending from the subglottis to the upper trachea.

A 61-year-old woman was admitted to our hospital with a three-week history of hoarseness and mild dyspnea. Her medical history included depression, for which she was taking milnacipran hydrochloride, olanzapine, flunitrazepam, and cloxazolam (antidepressants and sedatives). There was no history of fever, no night sweats, no weight loss, and no superficial lymphadenopathy, nor was there any history of tobacco or ethanol usage. Physical examination revealed normal vital signs. Laboratory data were largely unremarkable (white blood cells: 4.3×10^3^/μL, platelets: 371×10^3^/μL, hemoglobin: 11.1 g/dL, soluble IL-2 receptor: 433 U/mL, aspartate aminotransferase (AST): 79 U/L, alanine aminotransferase (ALT): 124 U/L, alkaline phosphatase (ALP): 349 U/L, and lactate dehydrogenase (LDH): 393 IU/L). No specific antibody testing for human papillomavirus (HPV), Epstein-Barr virus (EBV), or other relevant viral infections was performed; however, the patient had no history of symptomatic infections, including infectious mononucleosis, no relevant family or occupational history, and no prior HPV vaccination. The LDH elevation was modest, unlike that seen in typical aggressive lymphomas. The initial fiberscopic examination showed only mild edema around the vocal cords and no apparent subglottic lesion (Figure 1a), leading to a presumptive diagnosis of laryngitis and treatment with inhaled steroids as an outpatient. However, her symptoms worsened despite treatment (Figure 1b), and after four weeks she developed nocturnal dyspnea and presented to the emergency room with stridor (Figure 1c). Fiberscopic examination revealed multiple tumor lesions below the vocal cords, resulting in airway narrowing. Therefore, an emergent tracheostomy was performed by the otolaryngology team to secure the airway in the face of progressive obstruction. Contrast-enhanced CT (CECT) scans showed an iso-dense area involving the whole circumference of the trachea, which ranged from the subglottis to the proximal trachea (Figures 2a-2c). T2-weighted MRI scans showed the bulged posterior tracheal wall and an iso-intensity region around the trachea, which ranged from the subglottis to the proximal trachea (Figures 2d-2f). Fiberscopic examination through the tracheostomy stoma revealed multiple tumors on the anterior tracheal wall (white arrows) and a submucosal bulge on the posterior tracheal wall (blue arrows) in the subglottic area (Figures 1d, 1e); therefore, biopsies were taken from both areas. Because the initial biopsy had revealed only inflammatory changes, an open biopsy under general anesthesia was performed. Using forceps, the domed lesion on the dorsal side was excised, and several anterior lesions were also collected.

**Figure 3:**
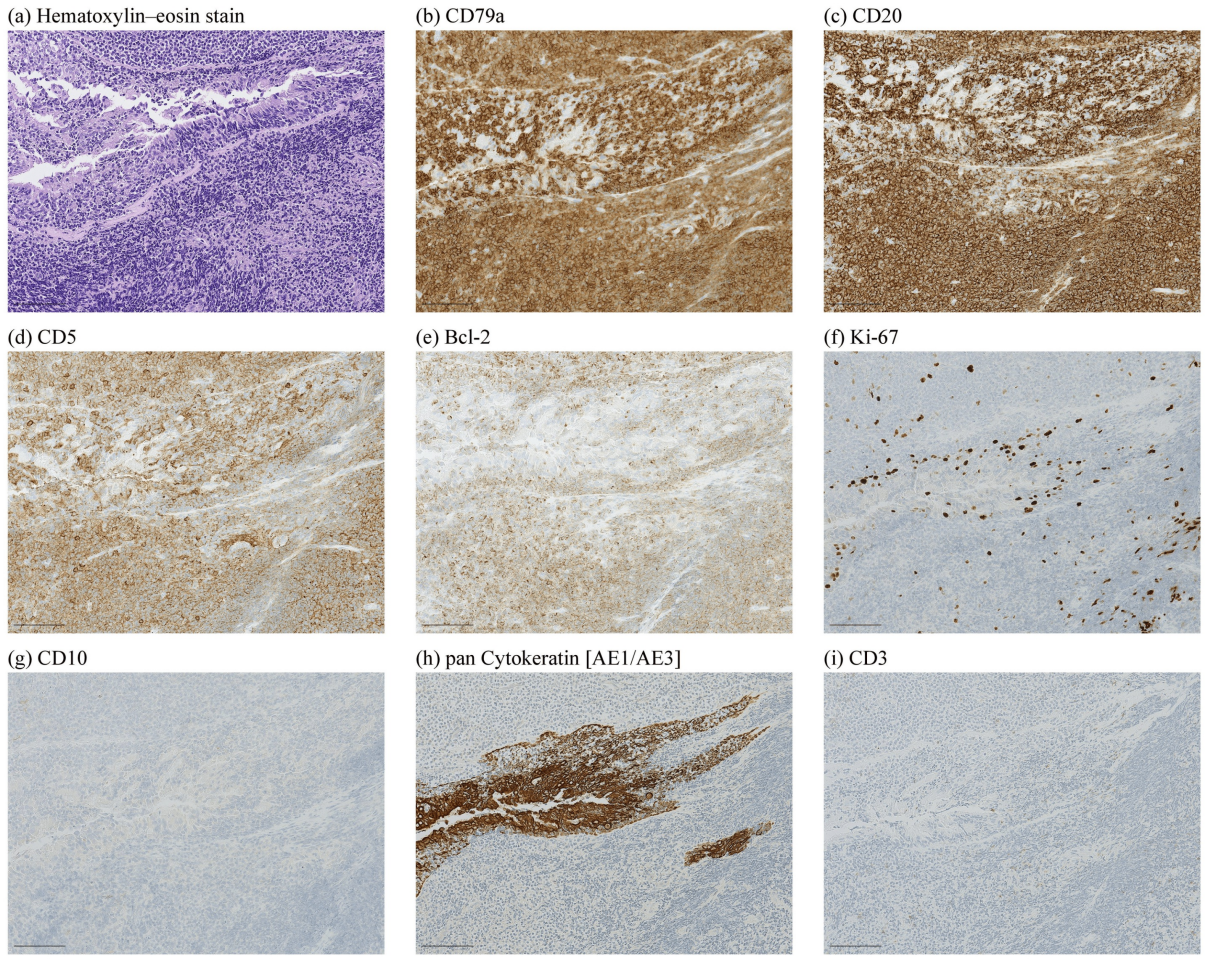
Microscopic images of the tumor (a) Hematoxylin and eosin stain; (b) CD79a; (c) CD20; (d) CD5; (e) Bcl-2; (f) Ki-67; (g) CD10; (h) Pan-cytokeratin (AE1/AE3); (i) CD3; scale bar: 100 µm.

Pathological findings showed diffuse small lymphoid cells invading subepithelium with lymphoepithelial lesion (LEL), and immunohistochemical results showed CD3(-), CD5(+), CD10(-), CD20(+), MUM1(-), CD23(-), CD79a(+), LEF1(-), SOX11(-), Bcl2(+), Bcl6(weakly+), Ki67(10-30% +), and cyclin D1(-) in the LEL area (Figure 3). Due to insufficient sample volume for fluorescence in situ hybridization (FISH), the case was diagnosed as CD5-positive low-grade B-cell lymphoma, unclassifiable according to the 2017 revision of the World Health Organization (WHO) classification of lymphoma [24]. The diagnosis was made by an external hematopathologist specializing in lymphoma. Although some uncertainty remains, the histopathological findings and clinical course support the plausibility of a CD5-positive MALT lymphoma.

**Figure 4:**
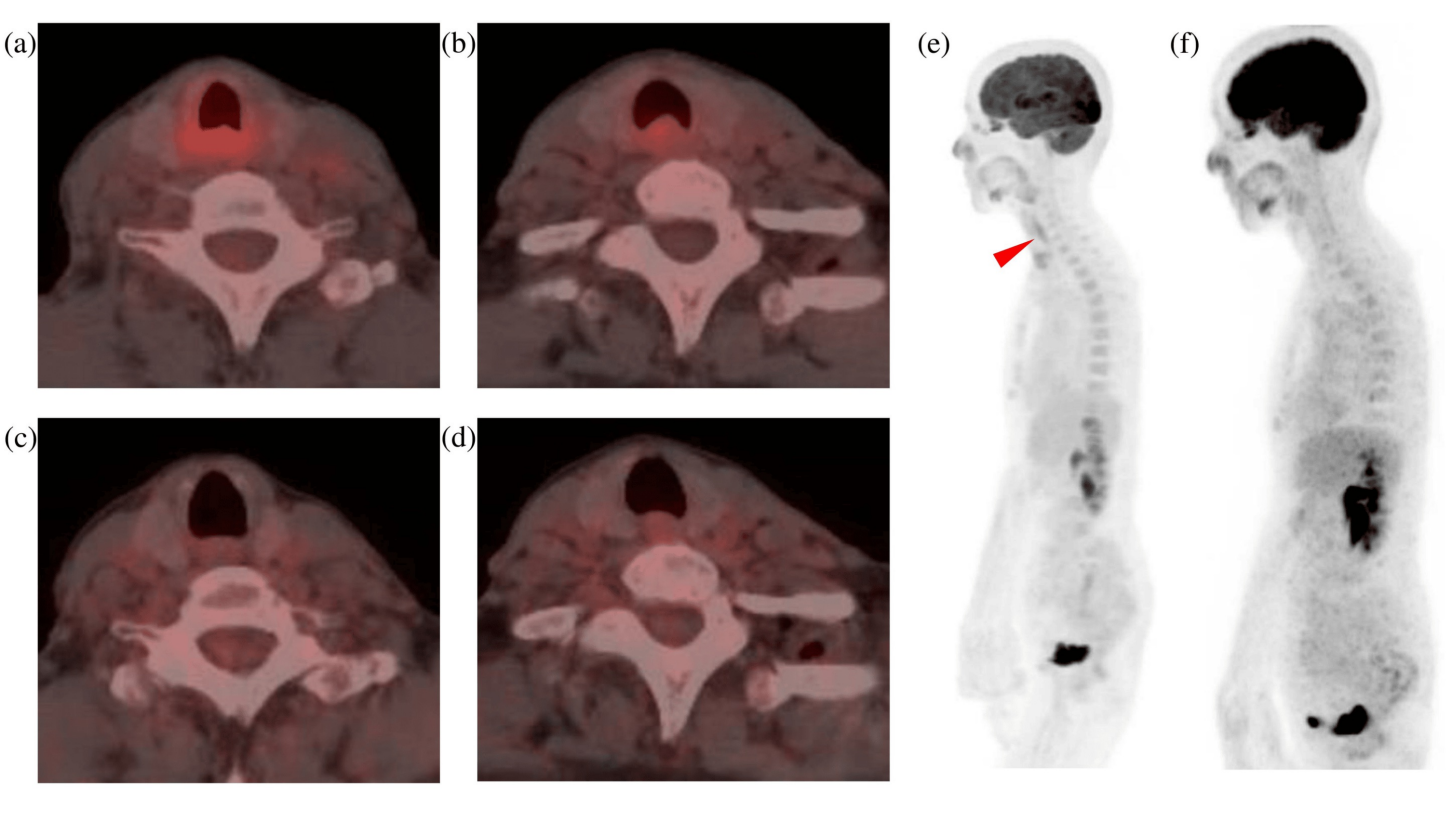
18F-FDG PET/CT images before and after four courses of chemotherapy (a,b,e) Before chemotherapy. The red arrow indicates increased FDG uptake in the posterior tracheal wall; (c,d,f) After chemotherapy FDG: fluorodeoxyglucose

^18^F-fluorodeoxyglucose (FDG) PET and CT scans showed increased FDG uptake in the bulged posterior tracheal wall, with a maximum standardized uptake value (SUV max) of 4.5, and no FDG uptake in the whole body without the bulged posterior tracheal wall (Figures 4a, 4b, 4e). Increased FDG uptake was also observed in both submandibular glands (maximum standardized uptake value (SUV max) 3.4), which was considered physiological uptake.

As a further biopsy would have required general anesthesia and might still have yielded insufficient tissue for definitive subclassification, treatment was initiated for localized low-grade B-cell lymphoma of unclassifiable type, with a plan to perform a repeat biopsy at recurrence for diagnostic confirmation. Three weeks after the tracheostomy, the patient was treated with combined chemotherapy with bendamustine and rituximab (BR) for four courses with an 80% dose reduction from the second cycle (bendamustine (day 1 and day 2): 120 mg/body (90 mg/m² (body surface area)) (first course) or 100 mg/body (72 mg/m² (body surface area)) (second, third, and fourth courses); rituximab (day 1): 520 mg/body (375 mg/m² (body surface area))) because of myelosuppression and liver damage, following discussion at a cancer board involving lymphoma specialists. After two courses of chemotherapy, the subglottic tumor and a bulge of the posterior tracheal wall disappeared (Figures 1f-1h), and her tracheostomy stoma was closed after improvement of the airway obstruction. The FDG uptake in the bulged posterior tracheal wall disappeared after four courses of bendamustine + rituximab (BR) chemotherapy (Figures 4c-4f). During the first year, the patient was seen in the otolaryngology clinic every three months, and thereafter, follow-up continued annually with both imaging and otolaryngology evaluations. After the treatment, her condition has remained stable for seven years.

## Discussion

The first symptom was hoarseness, reflecting the proximity of the lesion to the glottis. Within one month, dyspnea progressed and required emergent tracheostomy due to airway obstruction. The main initial symptoms of malignant lymphoma of the larynx have been reported to be hoarseness and difficulty breathing, and the main symptoms of malignant lymphoma of the trachea have been reported to include bloody sputum, cough, and dyspnea [25, 26]. In the glottis area, hoarseness appears when the lesion reaches the glottis; however, there may be no symptoms if the lesion is located above the vocal cords [4]. In subglottic malignant lymphoma, dyspnea is the most common symptom. While its progression is generally reported to be slow [27-30], dyspnea tends to appear earlier than hoarseness, reflecting the proximity of the lesion to the vocal cords. However, cases of Burkitt lymphoma [4] and some cases of MALT lymphoma [31] have been reported to show an aggressive course and can cause airway obstruction, requiring prompt diagnosis and treatment.

Macroscopically, the subglottic lymphoma in this case presented as a smooth submucosal bulge on the posterior side, accompanied by multiple papillary nodular lesions with similar characteristics on the anterior side. This is consistent with previously reported macroscopic characteristics of laryngeal lymphoma, which may appear as smooth mucosal swellings or multiple papillary nodular lesions [26, 32].

In terms of imaging, ^18^F-FDG-PET/CT revealed an area of nodular soft-tissue density in the dorsal subglottic tracheal area. Although low-grade MALT lymphomas typically show low FDG uptake, this lesion demonstrated moderate uptake (SUV 4.5), reflecting diagnostic uncertainty and highlighting the need for histopathological confirmation. A number of studies have shown that FDG uptake in MALT lymphoma depends on the disease site, and the FDG uptake was lower in low-grade than in aggressive lymphoma [33]. In some types of malignant lymphoma, including marginal zone lymphoma, there is no uptake of ^18^F-FDG [34, 35]. The avidity of FDG has been reported to be 100% in marginal zone lymphoma (nodal), 54%-81% in MALT marginal zone lymphoma, and 67% in marginal zone lymphoma (unspecified) [34].

Imaging findings of subglottic lymphoma can overlap with other causes of airway narrowing, such as amyloidosis [36-38], granulomatosis with polyangiitis (GPA) [39], or idiopathic subglottic stenosis [40]. Unlike amyloidosis, which often shows well-defined submucosal masses with calcifications, or GPA, which may demonstrate ulceration and cartilage destruction, laryngeal lymphoma typically presents as a uniformly enhancing soft-tissue lesion without necrosis or calcification. Routine laboratory studies are often nonspecific or normal, emphasizing that diagnosis relies primarily on imaging and histopathological evaluation rather than serum findings.

This case was diagnosed as low-grade B-cell lymphoma because further genetic diagnosis could not be performed due to insufficient sample volume. Low-grade lymphomas, such as MALT lymphoma, often exhibit minimal cytologic atypia. Sufficient high-quality tissue is required for immunohistochemistry and molecular analyses (e.g., FISH or gene rearrangement studies); however, clinical constraints may prevent adequate sampling, resulting in an unclassifiable diagnosis [22, 41]. The presence of lymphoepithelial lesions in the histological findings suggested MALT lymphoma. Based on the results obtained, the differential diagnosis for this case included CD5-positive MALT lymphoma, cyclin D1-negative mantle cell lymphoma (MCL) [42], or chronic lymphocytic leukemia (CLL). The CLL was excluded based on the clinical course. CD5-positive extranodal marginal zone (MALT) lymphoma is very rare, with reported primary sites including the cervical, axillary, and gastroepiploic lymph nodes [43], as well as the ocular region [44], whereas no cases originating in the larynx have been reported. Although CD5-positive cases may show a greater tendency to disseminate, several series report overall favorable, indolent clinical courses with good long-term survival when appropriately treated [44, 45].

Regarding treatment, primary laryngeal lymphoma is known to be sensitive to radiation, chemotherapy, or combined radiation and chemotherapy, and there is no difference in survival among these options [1]. Low-grade MALT lymphoma is sensitive to various therapeutic approaches, including radiotherapy, chemotherapy, and combined radiotherapy and chemotherapy [1]. *Helicobacter pylori* (*H. pylori*) eradication is added if the infection is detected [46]. Chemotherapy with bendamustine plus rituximab (BR) as a first-line treatment for primary or relapsed indolent non-Hodgkin's lymphoma (NHL) or mantle cell lymphoma (MCL) has been reported to be non-inferior to rituximab, cyclophosphamide, doxorubicin (hydroxydaunorubicin), vincristine (Oncovin), and prednisone (R-CHOP) standard therapy in terms of clinical response, with an acceptable safety profile [47-49]. BR therapy was selected based on prior evidence. The excellent efficacy of BR chemotherapy suggests that, despite its histological appearance of low-grade lymphoma, the tumor may have possessed biological features associated with higher chemosensitivity or an unrecognized aggressive component. Recommended post-treatment surveillance is multidisciplinary and individualized; guidance from major practice statements favors clinical review every three months for the first one to two years and then spacing visits to every six months thereafter, with imaging or site-directed endoscopy driven by symptoms or residual disease on baseline staging (PET/CT or CT as indicated) [50, 51].

Although MALT lymphoma generally follows an indolent clinical course, several reports have described atypical biological behavior or histopathological heterogeneity depending on the anatomical site and immunophenotype, particularly in CD5-positive cases, which may display a higher propensity for dissemination or transformation to more aggressive subtypes [44, 45]. Therefore, the disease behavior in this case may differ from that of typical indolent MALT lymphoma, and the histopathological classification could potentially be distinct due to its unusual immunophenotypic profile and subglottic origin [22, 24, 31, 44].

## Conclusions

We present a rare case of low-grade B-cell lymphoma, presumed to be CD5-positive MALT lymphoma, in the subglottic region, which required emergent tracheostomy due to subglottic tracheal airway stenosis and was treated with BR chemotherapy. While the precise subtype could not be definitively confirmed, this case highlights the importance of considering lymphoma in the differential diagnosis of unexplained subglottic airway obstruction. Laryngeal lymphoma is extremely rare and often poses a diagnostic challenge, but it should be considered in the differential diagnosis of patients presenting with sputum, cough, dyspnea, or hoarseness. Early consideration of lymphoma in cases of unexplained subglottic stenosis may prevent airway compromise and enable timely initiation of appropriate therapy.
